# Closure of ventricular septal defect in children with trisomy 18: perioperative events and long-term survival

**DOI:** 10.1093/icvts/ivaf010

**Published:** 2025-01-24

**Authors:** Shintaro Nemoto, Kanta Kishi, Hayato Konishi, Akiyo Suzuki, Takahiro Katsumata, Noriyasu Ozaki, Yutaka Odanaka, Atsuko Ashida, Takamichi Uchiyama, Kenji Mine

**Affiliations:** Department of Thoracic and Cardiovascular Surgery, Osaka Medical and Pharmaceutical University, Takatsuki, Osaka, Japan; Department of Pediatrics, Osaka Medical and Pharmaceutical University, Takatsuki, Osaka, Japan; Department of Thoracic and Cardiovascular Surgery, Osaka Medical and Pharmaceutical University, Takatsuki, Osaka, Japan; Department of Thoracic and Cardiovascular Surgery, Osaka Medical and Pharmaceutical University, Takatsuki, Osaka, Japan; Department of Thoracic and Cardiovascular Surgery, Osaka Medical and Pharmaceutical University, Takatsuki, Osaka, Japan; Department of Pediatrics, Osaka Medical and Pharmaceutical University, Takatsuki, Osaka, Japan; Department of Pediatrics, Osaka Medical and Pharmaceutical University, Takatsuki, Osaka, Japan; Department of Pediatrics, Osaka Medical and Pharmaceutical University, Takatsuki, Osaka, Japan; Uchiyama Kids Clinic, Takatsuki, Osaka, Japan; Department of Pediatrics, Kansai Medical University, Hirakata, Osaka, Japan

**Keywords:** trisomy 18, pulmonary artery banding, ventricular septal defect closure, long-term survival

## Abstract

**OBJECTIVES:**

This retrospective study aimed to investigate the feasibility of surgical closure of ventricular septal defect in children with trisomy 18 by assessing perioperative events and long-term survival.

**METHODS:**

From April 2008 to March 2024, 41 consecutive patients were referred to us for ventricular septal defect surgery. The defect was closed in 35 patients at the end (median age, 16 months; median body weight, 5.7 kg), 31 out of 37 patients after the preceding pulmonary artery banding according to our staged surgery policy and 4 patients without the banding. Sixty-five significant non-cardiac lesions existed concurrently, and 14 patients underwent tracheostomy before closure. The investigation was conducted by checking medical records and contacting the primary physician.

**RESULTS:**

Four patients died during the inter-stage after banding to closure (10.8%). Two of them were awaiting closure. Concomitant surgeries, 15 right ventricular muscle resections, or 1 arch repair, were performed along with closure. Arrhythmia was the most common adverse event (51.4%). Three patients required extracorporeal membrane oxygenation support. Transient but severe hepatic injury occurred in 11 patients (31.4%). There were two hospital death (5.7%) due to severe respiratory insufficiency or fulminant sepsis. Five patients died after discharge, three pneumonia and two sudden death, resulting in a 5-year estimated survival of 79.5%. Three hepatoblastoma and one hepatoangioma developed, but complete remission was achieved in all patients.

**CONCLUSIONS:**

Although further studies are mandatory, surgical closure of ventricular septal defect may be an effective treatment option even for children with trisomy 18.

## Graphical abstract

**Figure:**
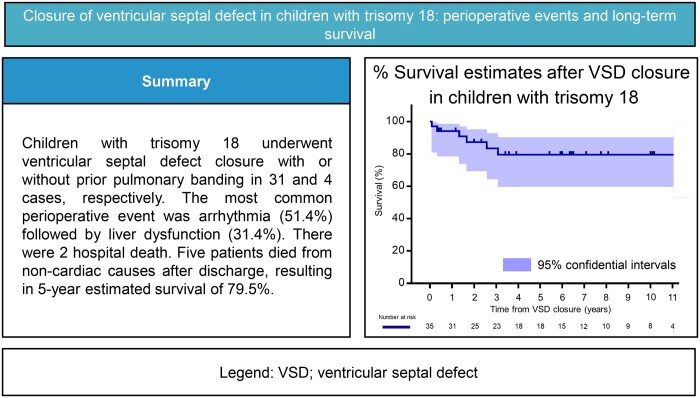

## INTRODUCTION

The increasing number of cohorts describing successful cardiac surgery for children with trisomy 18 (T18 children) sparks a new level of debate beyond ethics [1], such as guidance for decision-making [2]. The problem is that in most reports, long-term postoperative outcomes remain unclear, because cardiac surgery is performed without a clear distinction between palliative surgery and intracardiac repair by a consistent policy [3–5]. As a consequence, this uncertainty has led to unclear surgical results and wide differences in the attitudes of healthcare providers towards cardiac surgery among T18 children based on their specialties [6]. Furthermore, this confusion may also affect the information provided to patients’ families, which varies by institution, region, and nation.

This retrospective study was performed to add another evidence showing the current possible extent of cardiac repair in this vulnerable population by assessing the perioperative and long-term conditions of our consistent surgical policy, i.e. completion of intracardiac repair principally following palliative surgery for mild to moderate cardiac anomalies, i.e. ventricular septal defect (VSD).

## PATIENTS AND METHODS

### Ethical statement

The Institutional Review Board of Osaka Medical and Pharmaceutical University approved this retrospective observational study on 17 December 2024 (2024-197). The need for informed consent was waived by the IRB. Collection and storage of data from research participants for multiple and indefinite use strictly comply with requirements outlined in the WMA Declaration of Taipei. The IRB approved the establishment and continuously monitors ongoing use of the database.

### Surgical policy for patients with ventricular septal defects associated with trisomy 18

The feasibility of cardiac surgery was discussed by our multidisciplinary team on a case-by-case basis. An individual surgical plan was prepared to explain the procedure and possible risks of perioperative adverse events (AEs) and death to the patient’s family. Our liaison psychiatric nurse provided counselling upon request by the patient’s family. All parents provided informed consent for each cardiac operation.

Our goal is to establish normal circulation regardless of trisomy 18. Our challenge began with the establishment of surgical treatment for mild to moderate cardiac lesions, such as persistent ductus arteriosus, coarctation of the aorta, atrial septal defect, VSD, and tetralogy of Fallot, but not for severe complexed heart diseases, such as functional univentricular heart and complex cardiac anomalies, with relatively high surgical morbidity and mortality rates, even in patients without chromosomal abnormalities. This study only focused on the surgical treatment of VSD.

Because T18 children are generally fragile with multiple severe comorbid non-cardiac lesions, staged surgery, i.e. VSD closure following pulmonary artery banding (PAB), is our principal policy. PAB allows time for patient growth and parents’ decision-making regarding VSD closure. On the other hand, VSD closure was the first choice in cases of late surgical referral in which the timing of PAB was missed or in cases in which prompt VSD closure was required.

In principle, cardiac catheterization was performed before VSD closure to predict the occurrence and extent of perioperative pulmonary arterial hypertension (PAH), that is well known as a significant risk factor for morbidity and mortality in T18 children [7].

### Ventricular septal defect closure in children with tracheostomy

The tracheostomy tube was replaced with an oral endotracheal tube after induction of anaesthesia (Supplementary Material, Fig. A). A T-shaped sternotomy was performed to expose the heart and preserve the sternal manubrium (Supplementary Material, Fig. B). Under cardiopulmonary bypass and cardioplegic arrest, the VSD was closed with a synthetic patch, and the main pulmonary artery was reconstructed by removing the PAB band along with direct end-to-end anastomosis or patch augmentation. Inter-atrial communication with an 3-mm diameter was maintained in case of perioperative right ventricular dysfunction or PAH crisis. The tracheostomy tube was placed on the postoperative day (POD) 2 or 3.

### Patient inclusion

Consecutive T18 children who underwent cardiac surgery for VSD, either PAB or VSD closure, between 1 April 2008, and 31 March 2024 were included in this study.

### Data collection and outcome

Clinical course, preoperative and perioperative categorical variables and covariates, cardiac catheterization data, performed surgical procedures, and postoperative death and AEs during hospital stay were collected from the medical records of our institution. Information on the occurrence of deaths and AEs after discharge by the end of the study was obtained from the outpatient medical records or by contacting paediatricians who provided postoperative follow-up.

Outcomes were defined as death from any cause and severe AEs associated with a risk of death unless appropriate treatment.

### Statistics

Because of the nature of this retrospective study with a limited number of cases and ethically in a one-group setting, we used descriptive statistics to provide the overall clinical characteristics of VSD closure in T18 children. Descriptive data were expressed as median (interquartile range: IQR) or mean (standard deviation), as appropriate. On the other hand, Kaplan–Meier estimation was used to assess long-term survival after VSD closure using JMP Pro 16 statistic software (SAS Institute Inc., Cary, NC, USA).

## RESULTS

### Clinical flow of children advancing to ventricular septal defect closure from pulmonary artery banding

**Figure 1:**
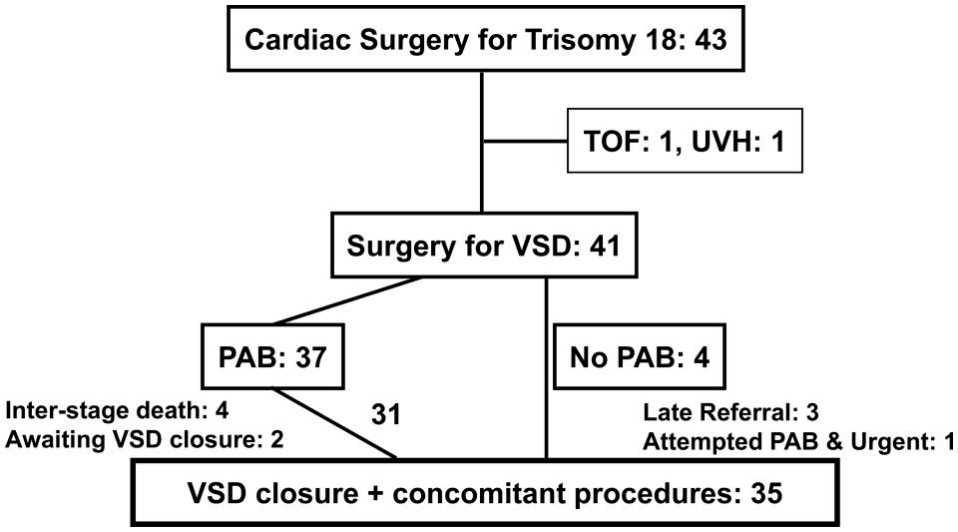
Clinical flow of children with trisomy 18 advancing to ventricular septal defect closure following pulmonary artery banding. PAB: pulmonary artery banding; TOF: tetralogy of Fallot; UVH: univentricular heart; VSD: ventricular septal defect.

During the 16 years of the study period, 43 T18 children underwent cardiac surgery at our institution (Fig. 1). Excluding one case each tetralogy of Fallot and univentricular heart, 41 consecutive cases underwent cardiac surgery for VSD. Thirty-seven of 41 patients underwent PAB, with a median age of 68 days (IQR 35–88) and a median bodyweight of 2.1 kg (IQR 1.8–2.5). Among them, 20 patients were referred for VSD closure after PAB from other institutions. While two patients were waiting for VSD closure at the end of the study period, four patients died at a median of 7 months (IQR 4–14) after PAB due to two severe infection, one complete atrioventricular block, and one post-resuscitation encephalopathy. In total, 31 patients achieved VSD closure after PAB with a median interval of 15.0 months (IQR 8–20.5).

In contrast, VSD closure was performed without PAB in three cases who were referred for surgery late at ages 7, 10 and 27 months from other institutions, and in a 4-month-old patient with severe hypoxaemia that required abandonment of PAB during surgery and persisted postoperatively. A total of 35 children underwent VSD closure through these two pathways, with a median age of 15 months (IQR 10.0–21.5) and a median body weight of 6.0 kg (IQR 4.9–6.5).

### Background of 35 children who underwent ventricular septal defect closure

Thirty of 35 patients (85.7%) were female. There was one case of complex VSD with coarctation of the aorta that underwent total correction following bilateral PAB. Concomitant right ventricular outflow muscle resection was performed in 15 patients. Eleven babies were born with extremely low birth weights. Table 1 shows a wide variety of comorbid non-cardiac lesions. The following surgical interventions were performed prior to VSD closure for the purpose of survival, physical strength, and safe management after open-heart surgery: 2 surgeries for central nervous system lesions, 3 oesophageal atresia repair, 8 surgeries for various digestive system lesions, 1 hepatoblastoma resection and 14 tracheostomies.

**Table 1:** Comorbid non-cardiac lesions in 35 children with trisomy 18 who underwent ventricular septal defect closure

	*N*	Surgery
Central nervous system^a^	4	
Brain abscess	1	Drainage
Myelomeningocele	1	Dural sac closure
Facial nerve palsy	1	
Hypoxic encephalopathy	1	
Respiratory^b^	25	Tracheostomy: before VSD closure 14 and after VSD closure 4
Laryngomalacia	3	
Tracheomalacia	7	
Bronchomalacia	4	
Tracheobronchomalacia	2	
Upper airway stenosis	1	
Vocal cord stenosis	1	
Ventilator dependence	4	
Obstructive chronic lung disease	2	
Aspiration pneumonia	2	
Chronic respiratory insufficiency	3	
Apnoea	4	
Oesophageal atresia	3	Repair 3
Digestive system^b^ (decreasing order)	19	10^c^
Gastroesophageal reflux	8	
Inguinal hernia	3	
Umbilical hernia	3	
Pyloric thickening (stenosis)	2	
Anal atresia	2	
Diaphragmatic hernia	2	
Obstructive ileus	2	
Others (one case each)	10	
Hepatic	5	Resection: before VSD closure 1, after VSD closure 2
Hepatoblastoma	4	
Hepatohemangioma	1	
Renal^b^	9	0
Horseshoe kidney	5	
Double renal pelvis	1	
Bladder diverticulum	1	
Others (no anomaly)	3	

a Excluding cerebellar hypoplasia, corpus callosum defect, epilepsy, hearing loss and central apnoea.

b Including duplicate.

c Before VSD closure 8 (one case each): pyloric muscle incision, fundoplication + gastrostomy, enterostomy, diaphragmatic hernia repair, umbilical intestinal resection + abdominal wall closure, anal atresia repair, release of ileus + hernia repair and small intestine resection.

After VSD closure 2 (one case each): fundoplication and inguinal hernia repair.

### Cardiac catheterization

Table 2 summarizes the cardiac catheterization data obtained from 25 of 31 patients (80.6%) after PAB and from 3 of 4 patients (75%) without PAB. Catheterization was not indicated in patients requiring an early surgery because of poor body weight gain and prolonged hospital stay despite PAB and unstable haemodynamics. Although a comparative study with or without PAB was not performed because of the large difference in the number of patients, PAB might protect the pulmonary circulation, resulting in mild PAH with mildly elevated pulmonary arteriole resistance. On the other hand, three patients without PAB showed moderate to severe PAH with persistently increased pulmonary blood flow. Since all patients showed positive responses on pulmonary vasodilation testing during catheterization, VSD closure was indicated. Patients without PAB tended to require postoperative PAH therapy, such as inhaled nitrous oxide or type 5 phosphodiesterase inhibitor.

**Table 2:** Cardiac catheterization data prior to ventricular septal defect closure

Parameters	Total 29/35 cases	
PAB (staged repair)	Yes	No
*N* % (case)	25/31 (80.6)	3/4 (75%)
Mean pulmonary artery pressure (mmHg)		
Mean (SD)	24 (7.2)	44 (5.0)
Median (min–max)	24 (12–45)	44 (38–50)
Mean blood pressure (mmHg)		
Mean (SD)	60.4 (12.3)	67.3 (23.3)
Pulmonary-systemic pressure ratio		
Mean (SD)	0.43 (0.13)	0.73 (0.22)
Median (min–max)	0.41 (0.25–0.78)	0.69 (0.54–0.98)
Pulmonary-systemic flow ratio		
Mean (SD)	1.46 (0.55)	2.43 (1.7)
Median (min–max)	1.3 (0.7–2.8)	1.85 (1.1–4.9)
Pulmonary artery resistance (Wood Unit·m^2^)		
Mean (SD)	3.49 (1.80)	7.43 (4.02)
Median (min–max)	3.5 (0.82–7.68)	7.45 (2.5–12.3)
RV/LV pressure ratio		
Mean (SD)	0.96 (0.06)	0.84 (0.25)
Median (min–max)	0.96 (0.84–1.18)	0.94 (0.46–1.0)
Perioperative PAH therapy	*N* % (case)	*N* % (case)
Inhaled nitrous oxide	3/31(9.7%)	1/4 (25%)^a^
Phosphodiesterase-V inhibitor	4/31 (12.9%)^a^	2/4 (50%)

a One perioperative death in each with/without PAB.

LV: left ventricle; PAB: pulmonary artery banding; PAH: pulmonary arterial hypertension; RV: right ventricle.

### Perioperative adverse events and mortality after ventricular septal defect closure

The perioperative AEs are summarized in Table 3. The most common AE was arrhythmia in 18 patients (51.4%), which later improved except 1 pacemaker implantation for complete atrioventricular block. A notable and second most common AE was transient but severe liver injury in 11 patients (31.4%). Severe AEs included sepsis in two patients (5.7%) and acute respiratory distress syndrome in one patient (2.9%). Three patients required extracorporeal membrane oxygenator (ECMO) support for severe right heart failure, not-resumption of heartbeat after release of aortic cross-clamp, and septic shock in one patient each. Two patients were weaned from ECMO, except for one patient with septic shock. Tracheostomy was required in four patients (11.4%) before discharge.

**Table 3:** Perioperative adverse events after ventricular septal defect closure

	*N*	% (out of 35 cases)
ECMO (weaned)	3 (2)	8.6
Delayed chest closure	3	8.6
Cardiac		
Severe left heart failure	2	5.7
Arrhythmia	18	51.4
Sinus dysfunction	4	
Atrioventricular block (pacemaker implantation)	5 (1)	
Atrial tachyarrhythmia	4	
Junctional ectopic tachycardia	2	
Ventricular tachyarrhythmia	3	
Respiratory		
Acute respiratory distress syndrome	1^a^	2.9
Tracheostomy after surgery	4	11.4
Non-cardiorespiratory		
Liver dysfunction	11	31.4
Sepsis	2^a^	5.7
Surgery-related		
Chylothorax	3	8.6
Phrenic nerve palsy	1	2.9
Re-exploration due to bleeding	1	2.9
Cardiac tamponade	1	2.9

a One perioperative death in each adverse even.

Two hospital deaths (5.7%) occurred. The patient was a 1-year-old girl who died on POD 30 due to fatal acute respiratory distress syndrome with massive bilateral pneumothorax despite successful weaning from ECMO for the sustained cardiac arrest mentioned above. This baby had an extremely low birth weight and underwent intestinal atresia surgery and PAB during the neonatal period. She had never been discharged from the referring institution and was referred to us for VSD closure as requested by her parents. The patient’s preoperative cardiac condition was fair, but her lung was severely emphysematous with interstitial proliferation due to chronic lung disease. The other patient was a 27-month-old girl who was diagnosed with VSD and mild right ventricular outflow stenosis and was referred to us from the same institution late in the clinical course without PAB. There were no severe non-cardiac lesions, and preoperative cardiac catheterization showed severe PAH but improved with pulmonary vasodilation testing. Therefore, VSD closure with right ventricular muscle resection was performed. Her postoperative course began stable with minimal pulmonary vasodilation therapy. She suddenly collapsed due to rapidly progressing fulminant septic shock on POD 4, and ECMO was initiated on POD 5 despite the various medical treatments. However, she was not weaned off from ECMO and died on POD 20.

The median ICU and hospital stay of 33 survivors were 9 days (IQR 5–15) and 32 days (IQR 22–49), respectively.

### Adverse events and mortality after hospital discharge and survival

Observational patient follow-up was discontinued on 16 April 2024. The median follow-up period after VSD closure in 35 patients was 65 months (IQR 24–97), and the longest was 172 months (14.3 years).

**Figure 2:**
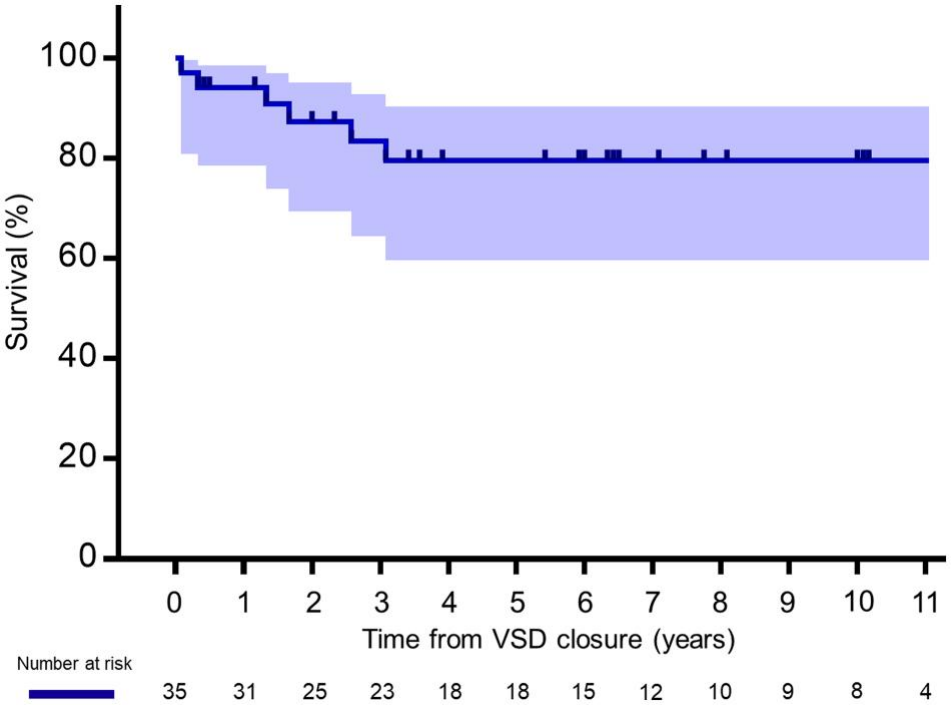
% Survival after ventricular septal defect closure in children with trisomy 18 determined using Kaplan–Meier survival estimates. The shaded area indicates 95% confidence intervals. VSD: ventricular septal defect.

All survivors (100%) were followed up by our team or the local paediatric cardiologists. Five patients died during follow-up: three due to pneumonia and two due to sudden death at 42, 50, 52, and 16, 52 months after VSD closure, respectively. Including the two hospital deaths, the 5-year survival rate after VSD closure was 79.5% (95% CI 59.6–90.3) calculated by Kaplan–Meier survival estimates, as shown in Fig. 2.

Of note, three hepatoblastoma and one hepatoangioma developed as severe AEs. All patients survived with complete remission following multidisciplinary treatment, as previously reported [8].

## DISCUSSION

The major findings of this retrospective study are as follows: first, PAB performed according to our two-staged surgery strategy, allowed not only ensuring physical strength to withstand VSD closure but also prevention of PAH even in T18 children as expected. However, the high frequency of death (10.8%) during the waiting period for VSD closure after PAB suggests the need for extra-cautious patient follow-up. Second, although T18 children were more likely to have a variety of comorbid non-cardiac lesions requiring prior surgeries, tracheostomy, or gastrostomy, these were not factors that disturb VSD closure. On the other hand, perioperative severe AEs relating to not only the cardiac but also the hepatic and respiratory systems occurred frequently. These might be the innate characteristics of trisomy 18. Third, although the selection criteria of patients for reliable surgical results remain unclear, VSD closure may offer a possibility of long-term survival for T18 children. Unfortunately, death due to severe infection or sudden death occurred within 5 years after discharge. Despite improvements in long-term survival, new problems, such as hepatic malignant tumour, have emerged.

Many reports to date have discussed the results of ‘cardiac surgery’ without distinguishing strictly between palliative surgery and anatomical repair [2–5], between trisomy 13 and trisomy 18 [2–7, 9–13], and between the different severities of cardiac anomalies [9]. Therefore, the outcomes of cardiac surgery in T18 children have not been clearly extracted. Therefore, the confusion caused difficulties in providing clear information about surgical interventions. To address this confusion, we made a first attempt to standardize the surgical strategy focusing on VSD, which is the most common and moderately severe cardiac anomaly in T18 children. To achieve invasive open-heart VSD closure, PAB is initially performed as palliative surgery whenever possible. This staged surgical procedure allowed successful VSD closure in 31 patients with less PAH at an appropriate time and weight for safe open-heart surgery. Coincidentally, correction of abnormal circulation is recommended at a moderate level for mild to moderate complexity, i.e. atrial septal defect, persistent ductus arteriosus, VSD, coarctation of the aorta, and tetralogy of Fallot, based on the American Association for Thoracic Surgery 2023 Expert Consensus document [12]. As initial palliative surgery is also recommended, our study results and those of previous reports [2, 9, 12–14] support the effectiveness of the staged approach. On the other hand, it should be noted that the mortality rate during the waiting period for VSD closure after PAB was as high as 10.8% (4/37 patients) and that the reported 5-year survival rate for PAB alone remained low at 18–35% [7, 14, 15]. If the parents expect the patient to survive long-term, it is important to proceed with VSD closure in a timely manner after placement of the PAB.

Most previous reports provide less detailed information about perioperative AEs after VSD closure for T18 children. This may leave the reality of VSD closure in this special population unclear and add unnecessary fear to primary physicians and patients’ families. An analysis from the Society of Thoracic Surgeons Database showed that the overall surgical mortality rate of 270 surgeries in T18 children was 15.6% (42 cases), and the incidence of AEs was 55.6% (150 cases) without distinction between surgical procedures and target diseases over an 8-year period [9]. The most common perioperative event in the Database was cardiac arrest in 18 patients (55.6%), followed by pacemaker implantation in 6 patients (2.2%), ECMO in 6 patients (2.2%), and unplanned intervention in 4 patients (1.5%). On the other hand, our patients experienced arrhythmia (51.4%), followed by liver dysfunction (31.4%), ECMO (8.6%), and sepsis (8.6%). Furthermore, in the database, preoperative mechanical ventilation was identified as a risk factor for perioperative death, whereas gastrostomy placement and prior palliative surgery contributed to survival. In contrast, although the number of cases was limited in our study, preoperative tracheostomy was not identified as a risk factor. Moreover, respiratory support through tracheostomy may help maintain the general condition until open-heart surgery in T18 children who often have tracheomalacia and/or bronchomalacia, as shown in Table 1. We agree that gastrostomy and prior palliative surgery improve survival after invasive open-heart surgery [9]. As several reports have indicated that treatment for airway complications is frequent after surgery in this population [2, 10, 14], we should add an explanation about these facts to the patient’s family when considering surgical treatment.

Our experience was consistent with that of contemporary reports examining 5-year survival rates after VSD closure varying from 66.6 to 83.9% [7, 11, 13, 15]. Furthermore, our findings coincide with the reported causes of death within 5 years after VSD closure, i.e. severe infection or sudden death [14]. On the other hand, it is noteworthy that there were no deaths from heart failure in the long-term, suggesting that the beneficial effects of VSD closure on cardiopulmonary function were achieved even in T18 children. This study revealed the risk of hepatic malignant tumour development as a fatal AE after VSD closure. Since long-term survival is now possible with cardiac surgery, the latent risk of the malignancy in T18 children may appear. Periodical screening using liver echo examinations and serum α-fetoprotein measurements become now our routine for early detection of tumours and appropriate treatment for successful remission, as recommended [16].

### Limitations

This retrospective study is based on descriptive statistics of only a limited number of patients who were conservatively selected for VSD closure at a single institution. In addition, this study did not perform any comparisons, such as surgical versus non-surgical cases, with or without PAB, or long-term survivors versus patients who died. Therefore, the factors that determine surgical success or failure remain unclear. Despite such constraints, we hope that the actual record in this study given by children born with trisomy 18 who strived for living with their beloved parents would help other families in need of cardiac surgery.

The treatment direction for T18 children is left to the decision-making of the family, but it is necessary to provide the latest and accurate information for this purpose. The data should not be strongly qualified by the fixed beliefs of the individual physician or institution. Unlike the recent trend of providing cardiac surgery for children with trisomy 21, not performing cardiac surgery is still an option for T18 children. However, we are no longer in a period when cardiac surgery should not be performed because of trisomy 18. It is necessary to present treatment options to families based on risk after evaluating each child’s condition, presence and severity of lesions and severity of heart disease and cardiopulmonary function. To help families of T18 children decide on a satisfactory treatment plan, medical professionals must continue to collect information on cardiac surgery for T18 children, conduct accurate evaluations, and disseminate that information for sharing.

### Conclusion

This retrospective study showed that VSD closure could contribute to long-term survival even in T18 children despite the high incidence of severe comorbid non-cardiac lesions and perioperative AEs. To ensure the small evidence in this study and clarify various unknown issues, it is mandatory to continue further research.

## Supplementary Material

ivaf010_Supplementary_Data

## Data Availability

The data related to this article will be shared upon reasonable request to the corresponding author.

## Notes

Presented at the 38th Annual Meeting of European Association of Cardio-Thoracic Surgery, Lisbon, Portugal, 9–12 October 2024.

## ABBREVIATIONS

AE: Adverse event
ECMO: Extracorporeal membrane oxygenator
IQR: Interquartile range
PAB: Pulmonary arterial banding
PAH: Pulmonary arterial hypertension
POD: Postoperative day
T18 children: Children with trisomy 18
VSD: Ventricular septal defect
