# Supplementary figures and images for: Closure of ventricular septal defect in children with trisomy 18: perioperative events and long-term survival
Source: Interdiscip Cardiovasc Thorac Surg. 2025 Jan 24;40(2):ivaf010. doi: 10.1093/icvts/ivaf010 (PMC11802472; doi:10.1093/icvts/ivaf010)

## Slide 1
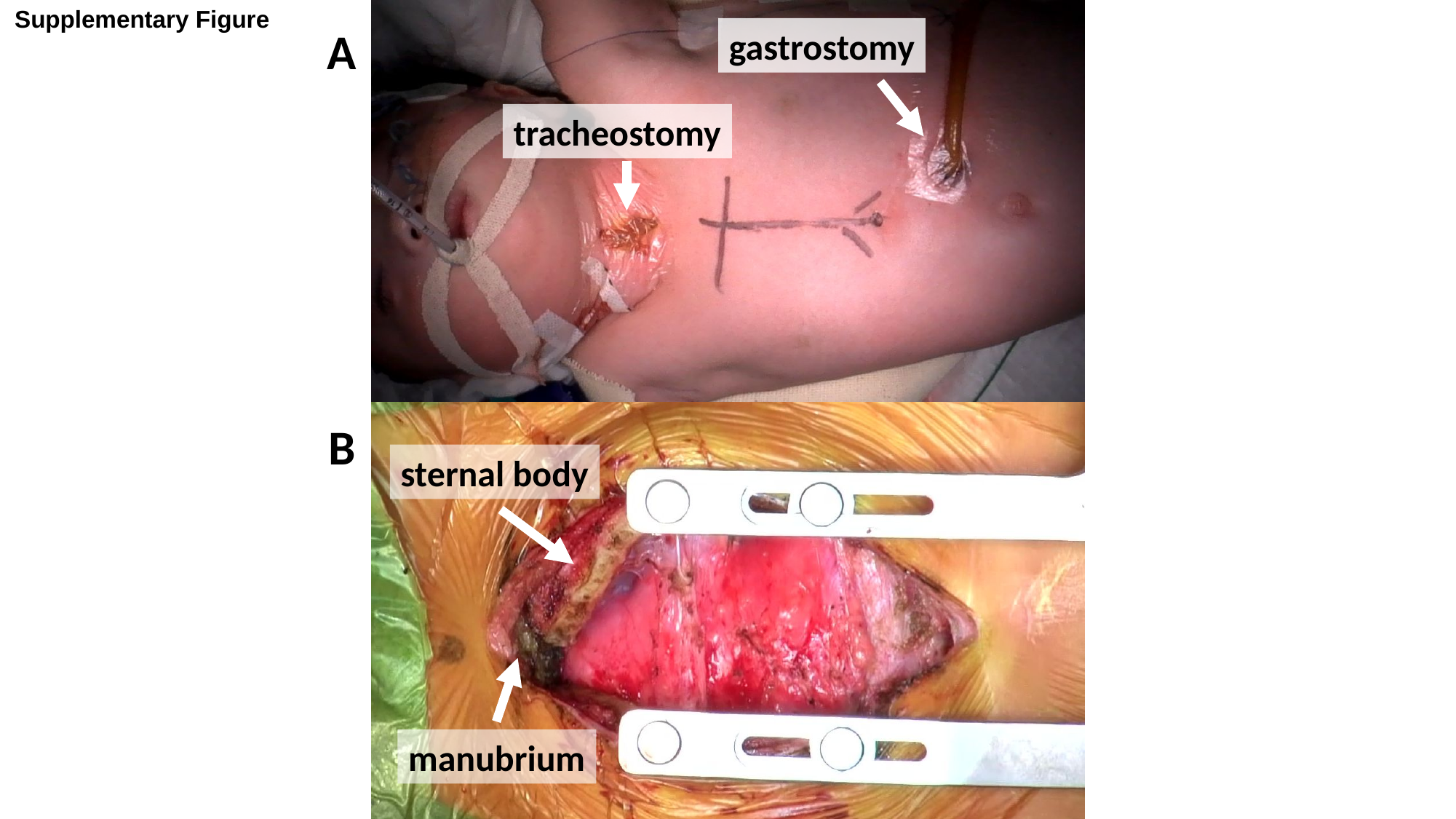

Supplementary Figure
A
gastrostomy
tracheostomy
B
sternal body
manubrium

Supplement: ivaf010_Supplementary_Data [file ivaf010_supplementary_data.pptx]
